# Does Participation in Physical Education Reduce Sedentary Behaviour in School and throughout the Day among Normal-Weight and Overweight-to-Obese Czech Children Aged 9–11 Years?

**DOI:** 10.3390/ijerph110101076

**Published:** 2014-01-16

**Authors:** Erik Sigmund, Dagmar Sigmundová, Zdenek Hamrik, Andrea Madarásová Gecková

**Affiliations:** 1 Center for Kinanthropology Research, Institute of Active Lifestyle, Faculty of Physical Culture, Palacký University in Olomouc, Tr. Miru 115, Olomouc 77111, Czech Republic; E-Mails: dagmar.sigmundova@upol.cz (D.S.); andrea.geckova@upjs.sk (A.M.G.); 2 Department of Recreation and Leisure Studies, Faculty of Physical Culture, Palacký University in Olomouc, Tr. Miru 115, Olomouc 77111, Czech Republic; E-Mail: zdenek.hamrik@upol.cz; 3 Health Psychology Unit, Institute of Public Health, Faculty of Medicine, P.J. Safarik University in Kosice 04011, Slovakia

**Keywords:** obesity, sedentary behaviour, physical education, physical activity, ActiTrainer accelerometer

## Abstract

Participation of 9 to 11-year-old children in physical education lessons (PEL) contributes to a significantly higher duration of moderate-to-vigorous physical activity (MVPA) during the school day and, in overweight/obese girls and normal-weight boys, to an increase in overall daily MVPA as shown by previous research. However, it is not known whether this increase in MVPA is at the expense of light physical activity (LPA) or sedentary behaviour (SED). SED, LPA, and MVPA were assessed in 338 schoolchildren aged 9–11 years (50.3% girls; 29.6% overweight/obese) over two school days (with and without a PEL) using a triaxial accelerometer during various segments of the school day. SED, LPA, and MVPA were quantified based on the duration of the activity (minutes). Participation in PEL led to significantly higher school MVPA in the overweight/obese and normal-weight girls and boys (*p* < 0.005) compared to MVPA of those children on the school day without PEL. Participation in PEL led to a significantly higher overall daily MVPA duration compared to that during the day without PEL for the overweight/obese girls (*p* < 0.05), normal-weight girls (*p* < 0.05) and boys (*p* < 0.005). Participation in PEL contributed not only to significantly higher LPA in the normal-weight girls and boys (*p* < 0.01) during the school day but also reduced school-time SED in the overweight/obese children (*p* < 0.01) and normal-weight girls (*p* < 0.005). Moreover, participation in PEL significantly reduced the overall daily SED in the normal-weight children and overweight/obese boys (*p* < 0.05). Adding one PEL to the daily school routine appears to be a promising strategy for effectively reducing SED in children.

## 1. Introduction

Sedentary behaviour (SED) is characterised as any waking activity that requires very low energy expenditure (≤1.5 METs) and that occurs while sitting or reclining [1,2,3]. A high amount of daily SED in school-aged children is associated with a higher risk of adverse health outcomes [2,4,5], including overweight and obesity [6,7] and lower fitness [8,9,10], regardless of the level of physical activity (PA) [8,9,11]. However, other studies confirm that a higher level of moderate-to-vigorous physical activity (MVPA) in children is associated with a reduced cardiometabolic risk irrespective of the amount of SED [12,13]. In addition, it should be noted that there is often little association between SED and PA [6,11]. A high level of SED and a high level of PA are not mutually exclusive behaviours [6], and it is possible for an individual to accumulate large amounts of both SED and PA throughout the day [3,6]. In other words, these findings emphasise the theory that too much SED and too little PA represent separate and distinct health risk factors. Further studies that would reveal the association between SED and PA across a large range of intensities based on objective monitoring of free-living behaviour in children are necessary [3,6].

The school environment offers structured time segments with formal (e.g., PEL, after-school clubs and care) and informal (e.g., school transport, recesses, lunch break) opportunities for PA to all children and adolescents regardless of their age, gender, socio-economic status, or body weight [14,15,16,17]. The school environment includes repeated changes in the levels of children’s behaviour, *i.e.*, SED, light PA (LPA), moderate PA, and vigorous PA [14,15,18,19]. In addition, longitudinal 2-year regular implementation of a higher amount of MVPA (from 1,718 to 3,247 steps per day and from 2.1 to 3.6 kcal/kg per day) in PEL, recess, and after-school care significantly (*p* < 0.005) contributes to an effective reduction of overweight and obesity in 6 to 10-year-old children [16,17,20]. Although schools are attractive settings for PA participation of normal-weight and overweight/obese children, accelerometer-based data focusing on MVPA during specific segments of the school day, including PEL data, are limited [14,15,18]. While the health benefits of participation in PEL and school recesses have been reported (maintenance of MVPA, less time spent watching television, improved VO_2max_) [19,20,21,22], the most recent studies of accelerometer-based SED and PA levels in 9 to 14-year-old children during the segmented school day did not include PEL in the analysis [14,15] or the analysis was incomplete [18,19]. Moreover, little is known about the engagement of normal-weight and overweight/obese girls and boys in SED, LPA, and MVPA during PEL in relation to the school day and overall daily PA levels and SED.

Previous studies have revealed the contribution of MVPA during PEL [23] and school recesses [24] to school day and overall daily PA in 9 to 11-year-old overweight/obese and normal-weight children. However, the interaction between PA levels and SED during various segments of the school day in overweight/obese and normal-weight children has not been previously discussed [23,24]. Moreover, the question of whether an increase in daily MVPA in overweight/obese girls and normal-weight boys due to participation in PEL leads to a reduction in SED remains unanswered. Therefore, the primary aim of the present study was to investigate SED and PA levels in 9 to 11-year-old normal-weight and overweight/obese boys and girls during different segments of the school day (before school lessons, during PEL and other lessons, recesses, and after school). The specific objectives were the following: (i) to describe the differences in SED, LPA, and MVPA during specific segments of the school day (before school, during PEL and other lessons, recesses, and after school) in the normal-weight and overweight/obese girls and boys; (ii) to quantify the achievement of the current recommendation of 60 min of MVPA daily during a school day with participation in PEL, compared to the school day without PEL; and (iii) to examine the changes in SED and PA levels between the day with participation in PEL and the day without PEL.

## 2. Methods

### 2.1. Sample and School Routine

The study included a total of six primary schools: one from a city of over 100,000 inhabitants—Olomouc; four schools from smaller towns of 6,500 to 20,000 inhabitants—Hranice na Moravě, Staré Město u Uherského Hradiště, Polička, and Rýmařov; and one school from a village of Lutín with less than 3,500 inhabitants. The primary schools are located in four of the 14 regions in the Czech Republic: Pardubice Region, Olomouc Region, Zlín Region, and Moravian-Silesian Region. The selection of primary schools corresponded with the distribution of the urban-rural population in the Czech Republic [25]. The convenience sample of all six primary schools was based on uniformly implemented mandatory daily school routines, similar size, similar available sports and equipment (one large gymnasium, one smaller dancing hall with a fitness section, and an outdoor field), and a similar number of students (450 to 650). All students from grades three and four from the selected schools were included in the study. Children entering grade three are 8 to 9 years old, and children entering grade four are aged 9 to 10. The school routine on one of the two monitored days included one 45-minute PEL.

All of the participating girls and boys followed a mandatory daily school routine, including five school lessons, three short recesses, one longer break, and one lunchtime break. The data were collected in April and May of 2012.

School lessons started at 8:00 a.m., lasted 45 min and finished at 12:15 p.m. On one of the monitored days, all children participated in one 45-minute PEL with a similar content—physical activity games (e.g., tag, simplified versions of dodge-ball/floor-ball) and exercises with equipment (e.g., ball dribbling, catching, throwing at a target, skipping rope, small trampoline jumping) in the gym. One of the four school recesses lasted 20 min, while the others were 5 min long. During the 20 min break, the children were allowed to eat their own snacks or to play in their classroom under teacher supervision. The lunchtime break started at 12:15 p.m. and finished between 12:45 and 13:00 p.m. After lunch, the children either left school or stayed in an after-school programme. Because not all children stayed in the after-school programme, the time spent in the after-school programme was not included in the school time period.

### 2.2. Instruments and Measurements

The ActiTrainer (ActiTrainer^TM^, Pensacola, FL, USA) is a small and lightweight (8.6 cm × 3.3 cm × 1.5 cm; 53 g) accelerometer-based wearable device. It is a multi-functional device composed of a heart-rate monitor, tri-axis solid-state accelerometer, electronic pedometer, inclinometer, and ambient light sensor. The recording of data can be viewed on a built-in display; however, the display was covered during monitoring. When turned on, the ActiTrainer can monitor and continually store recorded data over a period of 7 days. The validity and reliability of the ActiTrainer-based SED and free-living energy expenditure in non-laboratory conditions was verified with 4- to 11-year-old children [26,27], and the feasibility of assessing PA patterns in children was verified in 2-day monitoring of 9- to 11-year-old normal-weight and overweight/obese Czech children [23] and 9- to 10-year-old Polish girls and boys [24].

The ActiTrainer accelerometer was positioned around the waist above the right knee, and continuously measured SED and PA levels in the children in 15 s epochs for the entire body-wearing time. The body-wearing time was calculated by subtracting non-wear time from 24 h. Similar to a previous study [28], non-wear time was defined as at least 60 consecutive minutes of zero counts, with an allowance for 1 to 2 min of counts between 0 and 100. Total minutes and the proportion of total time (to account for variations in the length of daily wear time among children) spent in each segment of the school day (before school lessons, during PEL and other lessons, recesses, and after school) were determined for SED (<100 counts per min (cpm)), LPA (100 to 2,296 cpm), and MVPA (≥2,296 cpm). The cut-off points for SED, LPA, and MVPA were determined based on previously published studies [24,26,29,30,31]. Participation in PEL was controlled by periods of consecutive zero counts. Daily ActiTrainer records with periods of ≥2 min of consecutive zero counts during PEL were excluded from the data analysis.

The anthropometric characteristics of children were determined in advance before monitoring to prepare the individual PA log book for each participant. One week before the start of monitoring, parents were asked to provide information about the body height and weight of their children with 0.5-cm and 0.1-kg accuracy. The chronological age was calculated from the date of birth until the first monitoring day. The BMI (kg/m^2^) was calculated as the body weight (kg) divided by the body height (m) squared. Obese, overweight, and normal body mass were classified using percentile BMI charts for girls and boys between the ages of 5 and 19 [32], where overweight and obesity represented the 85–97% and >97%, respectively, on age-differentiated BMI charts available on the WHO website [32].

After completing morning hygiene routines on the first monitored day, the parents of the participating children fastened the elastic waist belt with the ActiTrainer tightly to the children’s right hip. After arriving at school, a study author and the teacher checked the functioning of the ActiTrainer and wrote down the time of arrival in the individual PA log book. The personalised individual PA log book included the chronological structure of the day according to the current school schedule to record the time of morning attachment of the device, arrival at school, beginnings and ends of lessons and recesses, departure from school, and evening removal of the device. Under the supervision of their class teachers, the participating children further recorded the beginning times of the school lessons and recesses in the individual PA log book. In the evening, the parents recorded the time when the elastic waist belt was removed.

### 2.3. Statistical Data Processing and Interpretation

All analyses were carried out using the SPSS v19.0 software (IBM SPSS, Inc., Chicago, IL, USA) and STATISTICA v.9 (StatSoft CR, Prague, Czech Republic). The data were analysed in total for all classes because the TwoStep cluster analysis found no indicator for clustering by school. A series of non-parametric Mann-Whitney U-tests were conducted to examine the differences in the dependent variables (before school lessons, during PEL and other lessons, recesses, and after-school time) and ActiTrainer-based time duration of SED, LPA, and MVPA in the normal-weight and overweight/obese girls and boys (separately). A comparison of anthropometric characteristics in the normal-weight and overweight/obese children was performed using the non-parametric Mann-Whitney U-test. The Wilcoxon pair test was repeatedly used to determine the differences in the duration of SED, LPA, and MVPA in each segment of the school day on the days both with and without PEL between the normal-weight and overweight/obese girls and boys. The Wilcoxon pair test was used to examine whether the normal-weight and overweight/obese boys and girls met the current recommendations of 60 min of MVPA daily [33,34,35] on the school day with participation in PEL compared to the day without PEL. The *p*-values < 0.05 (<0.01 and <0.005) were considered statistically significant. The strength of the relationship between the independent (time duration of SED, LPA, and MVPA in the normal-weight and overweight/obese girls and boys) and dependent (segments of the school day) variables was assessed by means of the effect size coefficient “*d*” for the non-parametric Mann-Whitney U-test and Wilcoxon pair tests [36,37]. The *d* values of 0.2, 0.5, and 0.8 can be interpreted as small, medium, and large effects [38].

### 2.4. Ethics

The Ethical Committee of the Faculty of Physical Culture, Palacky University in Olomouc approved the study. The children’s parents, their teachers, and school management representatives were informed of the objectives of this descriptive research survey. Written informed consent was obtained from the children’s parents, as was verbal consent from the children prior to any measurement procedures. All children and their parents participated in the study voluntarily and received no incentives.

## 3. Results

### 3.1. Sample Characteristics of the ActiTrainer-Based 2-Day Monitoring of SED and PA Levels

A total of 365 children (187 girls and 178 boys) between the ages of 9 and 11 years started the 3-day monitoring of overall daily sedentary behaviour and PA levels during the morning hours. The measurement on the first day was excluded from the data analysis because this recording was incomplete and the novelty of wearing the ActiTrainer device could have affected the initial activity (reactivity) [39]. The final ActiTrainer-based 2-day monitoring was completed, and a total of 338 children (170 girls and 168 boys) with a median age of 9.91 (range 9.34 to 10.50) years were included in the data analysis. An absence from all segments of the school routine or non-participation in PEL (periods of ≥2 min of consecutive zero counts) constituted a reason for excluding 10 and 17 children, respectively (representing 8% of girls and 6.7% of boys). All monitored children wore the ActiTrainer accelerometer continuously for 2 days (excluding sleeping, hygiene, and bathing times) for a minimum of 12 h per day. The ActiTrainer-based 2-day monitoring of overall daily sedentary time, light, and moderate-to-vigorous PA was conducted for a median value of 14.37 (range 12.31–16.36) h per day.

### 3.2. Sedentary Behaviour and PA Levels during Various Segments of the School Day

Table 1 and Table 2 show the descriptive characteristics, SED, and PA levels of the participating girls (Table 1) and boys (Table 2). Of all participating children, 24.7% of girls and 34.5% of boys were classified as overweight or obese. The overweight/obese girls did not significantly differ from their normal-weight classmates in the duration of SED, LPA, and MVPA in any segments of the school day (Table 1). The overweight/obese boys showed significantly lower SED in school recesses than did normal-weight boys. However, in any other segments of the school day, no significant difference in SED between the overweight/obese and normal-weight boys was found (Table 2). The overweight/obese boys had a significantly shorter MVPA duration after school and over the entire day compared to the normal-weight boys, but there were no significant differences in any of the other school time subcomponents (Table 2).

### 3.3. Achievement of Current Recommendation of 60 min of MVPA Daily

During the school day with participation in PEL, a significantly higher (*p* < 0.05, *d* = 0.73) percentage of the overweight/obese girls (+33.3; 47.6% *vs.* 14.3%) and a significantly (*p* = 0.05, *d* = 0.31) higher percentage of the normal-weight boys (+16.4; 47.3% *vs.* 30.9%) reached the recommended duration of 60 min of MVPA per day compared to the school day without PEL. Similarly, a higher percentage of the overweight/obese boys (+3.5; 20.7% *vs.* 17.2%; *d* = 0.09) and normal-weight girls (+3.1; 23.4% *vs.* 20.3%; *d* = 0.08) achieved 60 min of MVPA daily on the school day with PEL compared to the school day without PEL; however, these differences were not statistically significant.

**Table 1:** Anthropometric characteristics and duration of sedentary time and physical activity levels during 2-day monitoring (median (full ranges)) in overweight/obese and normal-weight girls.

Variable	Girls (*n* = 170)		
	Normal-weight (*n* = 128)	*d*	Overweight/obese (*n* = 42)
**Anthropometric data**			
Chronological age (years)	9.84 (11.18–8.69)	0.29	9.41 (11.21–8.58)
Body height (cm)	140.50 (159.00–125.00)	0.08	142.00 (150.00–131.00)
Body weight (kg)	31.00 (43.00–22.00)	1.33	43.00 (52.00–34.00) *
BMI (kg/m^2^)	15.79 (19.63–12.25)	1.48	21.22 (24.06–18.77) *
**SED**, time <100 counts per minute (min)			
Before school	13.39 (75.24–1.00)	0.04	13.38 (48.49–1.49)
In school	153.75 (238.21–75.00)	0.10	157.00 (220.75–107.21)
Physical education lesson	8.21 (17.00–1.25)	0.01	8.20 (10.00–2.25)
Other lessons	132.63 (182.25–67.25)	0.10	134.63 (196.50–78.75)
Recesses	16.25 (50.00–4.75)	0.25	18.13 (29.25–4.75)
After school	292.19 (577.28–128.68)	0.02	316.97 (495.72–119.51)
All day	477.31 (767.50–261.57)	0.08	502.69 (701.05–241.61)
**LPA**, time ≥100 and <2,296 counts per minute (min)			
Before school	24.99 (73.50–1.21)	0.11	23.75 (50.00–3.15)
In school	113.38 (198.25–68.50)	0.10	110.13 (197.50–57.25)
Physical education lesson	27.04 (36.25–20.50)	0.17	27.75 (34.50–21.25)
Other lessons	69.00 (157.25–26.50)	0.05	69.63 (127.75–27.00)
Recesses	32.75 (81.75–15.25)	0.20	31.50 (72.25–18.25)
After school	218.51 (359.14–1.00)	0.02	206.11 (319.66–16.97)
All day	360.92 (548.32–127.36)	0.12	353.98 (520.41–142.73)
**MVPA**, time ≥2,296 counts per minute (min)			
Before school	1.50 (18.75–0.00)	0.25	2.55 (19.50–0.07)
In school	10.00 (73.18–2.16)	0.08	11.65 (31.45–1.91)
Physical education lesson	9.95 (19.00–0.75)	0.04	9.95 (20.00–1.25)
Other lessons	2.30 (21.75–0.50)	0.02	1.84 (15.00–0.82)
Recesses	2.75 (59.80–0.50)	0.09	3.00 (15.00–0.00)
After school	26.89 (85.72–0.00)	0.10	25.96 (115.84–0.00)
All day	42.90 (112.23–5.49)	0.12	43.42 (139.58–8.91)

Notes: *n*, number of participants; BMI, body mass index; SED, sedentary behaviour; LPA, light physical activity; MVPA, moderate-to-vigorous physical activity; PEL, physical education lesson; *d*, effect size coefficients; statistical significance (Mann-Whitney test) for the differences between the normal-weight and overweight/obese girls * *p* < 0.001.

**Table 2:** Anthropometric characteristics and duration of sedentary time and physical activity levels during 2-day monitoring (median (full ranges)) in overweight/obese and normal-weight boys.

Variable	Boys (*n* = 168)		
	Normal-weight (*n* = 110)	*d*	Overweight/obese (*n* = 58)
**Anthropometric data**			
Chronological age (years)	9.99 (11.28–8.67)	0.09	10.04 (11.12–8.87)
Body height (cm)	142.00 (159.00–128.00)	0.56	148.00 (160.00–127.00) ***
Body weight (kg)	32.00 (45.00–22.00)	1.39	46.00 (70.00–30.00) ***
BMI (kg/m^2^)	15.87 (18.88–13.20)	1.62	20.41 (27.34–16.86) ***
**SED**, time <100 counts per minute (min)			
Before school	16.59 (56.25–0.75)	0.13	14.50 (43.15–0.50)
In school	140.38 (347.21–60.00)	0.21	139.38 (232.75–74.25)
Physical education lesson	7.50 (14.50–0.00)	0.02	7.17 (20.00–1.25)
Other lessons	122.13 (224.75–42.75)	0.17	116.50 (186.75–57.25)
Recesses	15.38 (44.25–1.75)	0.35	11.88 (48.00–3.25) *
After school	300.52 (614.23–127.00)	0.25	348.43 (567.47–110.71)
All day	465.16 (864.28–261.74)	0.15	487.22 (779.72–234.46)
**LPA**, time ≥100 and <2,296 counts per minute (min)			
Before school	24.50 (73.50–2.45)	0.23	30.87 (72.24–2.67)
In school	126.88 (230.58–51.00)	0.26	131.25 (211.98–21.50)
Physical education lesson	26.77 (38.00–13.75)	0.02	26.70 (35.75–19.25)
Other lessons	75.71 (171.75–25.00)	0.19	82.13 (152.75–18.00)
Recesses	33.50 (70.75–6.25)	0.28	35.88 (57.50–3.50)
After school	190.15 (320.93–14.50)	0.13	175.12 (375.77–4.51)
All day	341.02 (512.42–98.20)	0.02	337.76 (514.76–42.51)
**MVPA**, time ≥2,296 counts per minute (min)			
Before school	2.00 (24.75–0.00)	0.06	2.32 (15.75–0.07)
In school	14.32 (63.34–1.95)	0.24	9.15 (39.75–1.91)
Physical education lesson	10.12 (31.25–4.50)	0.11	9.95 (21.00–0.00)
Other lessons	3.00 (33.75–0.50)	0.21	1.97 (22.50–0.75)
Recesses	3.54 (31.25–0.50)	0.21	2.72 (27.25–0.82)
After school	31.40 (104.74–0.00)	0.40	16.83 (134.31–0.40) *
All day	52.42 (129.85–7.92)	0.45	34.68 (162.90–7.64) **

Notes: *n*, number of participants; BMI, body mass index; SED, sedentary behaviour; LPA, light physical activity; MVPA, moderate-to-vigorous physical activity; PEL, physical education lesson; *d*, effect size coefficients; statistical significance (Mann-Whitney test) for the differences between the normal-weight and overweight/obese girls * *p* < 0.05, ** *p* < 0.01, and *** *p* < 0.001.

### 3.4. Changes in SED and PA Levels between the Days with and without PEL

Participation in PEL led to significantly higher school MVPA in the overweight/obese and normal-weight girls and boys (*p* < 0.005) compared to MVPA of those children on the school day without PEL (Table 3 and Table 4). Participation in PEL led to a significantly higher overall daily MVPA duration compared to that during the day without PEL for the overweight/obese girls (*p* < 0.05, *d* = 0.67), normal-weight girls (*p* < 0.05, *d* = 0.55) and normal-weight boys (*p* < 0.005, *d* = 0.65).

**Table 3:** Comparison of duration (minutes) of school-day SED, LPA and MVPA (median) in overweight/obese and normal-weight girls between the day with participation in PEL and the day without PEL.

Variable	Girls (*n* = 170)					
	Normal-weight (*n* = 128)			Overweight/obese (*n* = 42)		
	Day with PEL	*d*	Day without PEL	Day with PEL	*d*	Day without PEL
**SED**						
Before school	13.87	0.05	13.39	13.39	0.29	12.75
In school	150.25	0.72	158.75 ***	154.53	0.78	162.25 **
After school	275.03	0.37	317.90	288.50	0.28	325.81
All day	460.42	0.53	480.70 *	484.33	0.41	512.54
**LPA**						
Before school	20.36	0.49	27.75	21.65	0.19	24.25
In school	119.39	0.75	108.00 ***	111.25	0.57	107.25
After school	212.05	0.11	223.51	202.74	0.41	207.98
All day	358.62	0.14	360.92	352.87	0.06	361.38
**MVPA**						
Before school	1.12	0.40	1.78	2.78	0.28	1.50
In school	14.90	1.70	4.82 ***	15.66	1.22	5.45 ***
After school	26.02	0.30	27.06	27.49	0.07	24.15
All day	44.19	0.55	36.27 *	49.29	0.67	35.90 *

Notes: *n*, number of participants; SED, sedentary behaviour; LPA, light physical activity; MVPA, moderate-to-vigorous physical activity; PEL, physical education lesson; *d*, effect size coefficients; statistical significance (Wilcoxon pair test) for the differences between the normal-weight and overweight/obese girls on the day with PEL and without PEL is expressed as * *p* < 0.05, ** *p* < 0.01, and *** *p* < 0.005.

**Table 4:** Comparison of duration (minutes) of school-day SED, LPA and MVPA (median) in overweight/obese and normal-weight boys between the day with participation in PEL and the day without PEL.

Variable	Boys (*n* = 168)					
	Normal-weight (*n* = 110)			Overweight/obese (*n* = 58)		
	Day with PEL	*d*	Day without PEL	Day with PEL	*d*	Day without PEL
**SED**						
Before school	16.66	0.25	16.50	13.00	0.11	15.50
In school	136.25	0.24	142.00	127.00	0.69	140.75 **
After school	282.62	0.49	324.53 *	314.99	0.49	364.18 *
All day	438.61	0.50	492.77 *	454.33	0.55	524.53 *
**LPA**						
Before school	21.75	0.07	25.10	26.50	0.59	34.25 *
In school	129.00	0.51	115.25 **	131.58	0.47	129.00
After school	191.28	0.07	186.99	173.33	0.07	182.11
All day	342.60	0.21	335.80	324.04	0.01	341.36
**MVPA**						
Before school	2.00	0.06	1.99	2.00	0.08	2.60
In school	20.00	1.13	6.59 ***	17.29	1.14	5.25 ***
After school	35.17	0.07	30.39	16.25	0.03	18.17
All day	59.79	0.65	40.98 ***	41.17	0.43	31.66

Notes: *n*, number of participants; SED, sedentary behaviour; LPA, light physical activity; MVPA, moderate-to-vigorous physical activity; PEL, physical education lesson; *d*, effect size coefficients; statistical significance (Wilcoxon pair test) for the differences between the normal-weight and overweight/obese boys on the day with PEL and without PEL is expressed as * *p* < 0.05, ** *p* < 0.01, and *** *p* < 0.005.

During the school day with participation in PEL the normal-weight girls (*p* < 0.005, *d* = 0.75) and normal-weight boys (*p* < 0.01, *d* = 0.51) achieved significantly higher LPA in school compared to the school day without PEL (Table 3 and Table 4). Participation in PEL led to significantly shorter school SED in the normal-weight girls (*p* < 0.005, *d* = 0.72), overweight/obese girls (*p* < 0.01, *d* = 0.78), and overweight/obese boys (*p* < 0.01, *d* = 0.69) compared to the school day without PEL (Table 3 and Table 4). Moreover, during the school day with participation in PEL, significantly shorter overall daily SED was observed in the normal-weight girls (*p* < 0.05, *d* = 0.37), normal-weight boys (*p* < 0.01, *d* = 0.50), and overweight/obese boys (*p* < 0.05, *d* = 0.55).

## 4. Discussion

The primary aim of this study was to investigate accelerometer-based SED and PA levels in the normal-weight and overweight/obese school-aged children during various segments of the school day (before school lessons, during PEL and other lessons, recesses, and after school). The evident specificity of this accelerometer-based monitoring of school and free-living behaviour in children was the inclusion of SED, LPA, and MVPA—for a minimum of 12 h per day over 15 s epochs. The findings of this study help bridge the gap between the current valuable publications from Western countries [14,15,18,19] and a lack of studies in Central and Eastern European countries (e.g., Czech Republic) by providing a detailed analysis of the differences in SED and MVPA duration during specific segments of the school day by gender and body mass in 9 to 11-year-old Czech children.

In relation to the first specific objective, we described and compared differences in the SED, LPA, and MVPA during specific segments of the school day in the normal-weight and overweight/obese girls and boys. Contrary to the results of previous accelerometer-based studies [40,41,42], we found no significant differences in SED, LPA, and MVPA between the overweight/obese girls and their normal-weight classmates during any segment of the school day. The normal-weight boys significantly exceeded the overweight/obese boys in MVPA only during after-school time and in the overall sum of daily activities. Previous studies have demonstrated that 8 to 12-year-old overweight/obese children spend substantially less MVPA time than normal-weight children during the morning and afternoon hours [37,40] and in reference to overall total daily activities [40,41,42]. Additionally, it has been shown that overweight/obese children engage less frequently in continuous MVPA lasting longer than 5, 10, or 20 min [41,42].

Our findings are unexpected but encouraging. The comparable level of accelerometer-based SED and PA levels in the normal-weight and overweight/obese children during school hours validate the study-design requirement for all participating children to undergo a daily school routine, including five school lessons, three short recesses, one longer break, and one lunchtime break. In addition, a similar duration of after-school SED in the normal-weight and overweight/obese children is likely associated with a dramatic increase in screen-based SED (TV/video viewing, playing PC/video games, searching the Internet, and chatting) in Czech adolescents in the last decade regardless of their weight status [7,43]. The independence of SED on weight status in 9 to 10-year-old children from Denmark, England, Estonia, Portugal, and Norway has also been confirmed in other accelerometer-based studies [18,44]. In accordance with previously mentioned studies [18,40,41], greater differences in MVPA and SED between normal-weight and overweight/obese children were identified in the after-school segment than in the in-school segment. The differences in after-school MVPA according to weight status are most overt when children are more likely to have the capability or motivation to choose to be active [41,45]. Overweight/obese children are less confident in their ability to overcome barriers to PA, ask their parents to provide opportunities for PA, or choose physically active pursuits over sedentary ones [42]. However, interpreting the differences in accelerometer-based SED, LPA, and MVPA between normal-weight and overweight/obese children from the after-school period is complicated due to a high degree of variability in day-to-day PA [40,46].

As for the second specific objective, we quantified the achievement of the current recommendation of 60 min of daily MVPA during the school day with participation in PEL in the normal-weight and overweight/obese girls and boys compared to that of a school day without PEL. While a comprehensive comparison of the results from different accelerometer-based studies may be difficult due to the differences in measurement and procedures among different countries [39,47,48,49], the current guidelines based on previous research and expert opinions suggest that at least 60 min of daily MVPA is required for youth to achieve optimum health benefits [33,34,35]. Despite the differences in various studies in the choice of epoch lengths and cut-off points of accelerometer counts to identify SED, LPA, and MVPA levels [29,50,51,52], paired comparison of the same normal-weight and overweight/obese children provides valid information about the observed phenomena. These observations are independent of the choices of epoch length or cut-off values for accelerometer counts.

In the present study, more than 47% of the overweight/obese girls and normal-weight boys achieved the recommended 60 min of daily MVPA during the school day with participation in a PE lesson. In contrast, only 14.3% of the overweight/obese girls and 30.9% of the normal-weight boys reached 60 min of MVPA on the day without PEL. Similarly, almost 21% of the overweight/obese boys and 24% of the normal-weight girls met the current daily MVPA recommendation during the school day with PEL unlike the day without PEL, in which only 17% of the overweight/obese boys and 20% of the normal-weight girls achieved the recommended MVPA targets.

With regard to the third specific objective of the current study, we examined the changes in SED and PA levels between the day with participation in PEL and the day without PEL. Previous school-day segmented accelerometer-based studies either did not analyse or inadequately analysed SED and PA levels during PEL [14,15,18,19]. The present study demonstrated that participation in PEL reduces school time SED in the overweight/obese children and normal-weight girls. Moreover, participation in PEL significantly reduces the overall daily SED in the normal-weight children and overweight/obese boys.

The monitored PEL of the participating children included a wide range of activities of various intensities, for example, SED (1-minute relaxation while lying), LPA (calisthenics, stretching, breathing exercises), and MVPA (e.g., dodge-ball/floor-ball, skipping rope, small trampoline jumping). The triaxial ActiTrainer accelerometer used in the study consists of three orthogonal accelerometer units and provides a valid estimate of these types of children’s PA [24,47,48]. The temporally highly transitory and intermittent PA-intensity pattern of children [50] throughout the school day allows the triaxial accelerometers to perform an objective assessment due to the short epoch lengths [23,47,48].

The school environment is an attractive setting for involving children with various levels of PA, which can contribute to the identification of health-relevant PA recommendations [20,23,24]. However, only long and repeated execution of relatively high amounts of MVPA in PEL, recess, and after-school care leads to an effective reduction of overweight and obesity incidence in 6 to 10-year-old children [16,17,20]. For example, meeting the NASPE recommended 100 min per week during recesses was associated with a 0.74 unit decrease in the BMI percentile for a large cohort of 6 to 11-year-old children in the UK [20]. Moreover, achieving the NASPE recommended 150 min per week in PEL was associated with a 1.56 unit decrease in BMI percentile among boys [20]. Objective accelerometer-based information of the distribution of SED, LPA, and MVPA in various segments of the school day and the contribution of participation in PEL to the reduction of overall daily SED could be beneficial in planning and implementing PA-enhancing programmes for children.

## 5. Limitations and Future Research

The limitations of this study include the study design (non-randomised controlled trial) and the relatively small sample size in the subcategories of normal weight and overweight/obese groups of children. Therefore, the conclusions require cautious generalisation with respect to extrapolating these results to a wider population of children in the Czech Republic. Furthermore, 2-day monitoring could have influenced the SED and PA levels of children due to reactivity, although 2-day pedometer monitoring of PA in 8 to 11-year-old children during and outside of school has been established as non-reactive [53,54]. The epoch lengths and cut-off points of accelerometer counts used in identifying SED and PA levels [29,51,52] could have influenced the classification of sedentary and MVPA time, but a pairwise comparison of the accelerometer-based SED and PA levels between the school days with and without PEL was not affected by these potential inaccuracies. A potential source of bias could be the fact that this study did not analyse the after-school PA programme, which could have been an important contributor to the overall daily MVPA in the normal weight and overweight/obese children [55]. Other potential confounders could include socio-economic status, parental education, and other family and home environmental characteristics not monitored within the study. Despite these limitations, the ActiTrainer-based 2-day monitoring of overall daily SED, LPA, and MVPA supports the conclusions that have arisen from pairwise comparison of SED and PA levels between school days with and without PEL.

For a more accurate assessment of SED and PA levels both during school time as well as throughout the entire day using the ActiTrainer accelerometer, a longer monitoring period (optimally seven consecutive days) [50,56] during various periods of the school year is necessary [18,57,58]. A more comprehensive understanding of SED and PA levels on a school day in school-aged children also requires valid information as to the possible influence of lifestyle behaviour—family and home-environmental characteristics, socio-economic status, parental behaviour, self-esteem, pro-social behaviour, and academic achievement [5,18,30,31]. Future research should also combine a global positioning system (GPS) with multi-functional accelerometer-based devices for studying PA levels patterns and SED of children [56]. Although this combination of methodological approaches brings technological and scientific advances, it must be balanced against the feasibility of the research [39].

## 6. Conclusions

The results of this accelerometer-based assessment of sedentary behaviour and physical activity levels provide important information about the effects of participation in physical education lessons on both school time and overall daily sedentary behaviour and physical activity levels in the normal-weight and overweight/obese children. Participation in physical education lessons led to significantly higher school moderate-to-vigorous physical activity in the overweight/obese and normal-weight girls and boys (*p* < 0.005) compared to moderate-to-vigorous physical activity of those children on the school day without physical education lessons. Participation in physical education lessons led to a significantly higher overall daily moderate-to-vigorous physical activity duration compared to that during the day without physical education lessons for the overweight/obese girls (*p* < 0.05), normal-weight girls (*p* < 0.05) and boys (*p* < 0.005). Participation in a physical education lesson contributes not only to a significantly higher level of light physical activity in 9 to 11-year-old normal-weight girls (*p* < 0.005) and normal-weight boys (*p* < 0.01) during school time but also reduces sedentary time in school in overweight/obese girls and boys (*p* < 0.01) and normal-weight girls (*p* < 0.005). Moreover, participation in a physical education lesson significantly reduces the overall daily sedentary time in normal-weight girls and overweight/obese boys (*p* < 0.05) and normal-weight boys (*p* < 0.01). During the school day with participation in a physical education lesson, a significantly (*p* < 0.05) higher percentage of the overweight/obese girls and normal-weight boys, and a non-significantly higher percentage of the normal-weight girls and overweight/obese boys achieved the recommended 60 min of moderate-to-vigorous physical activity compared to the school day without a physical education lesson. Adding one physical education lesson to the daily school routine appears to be a promising strategy for effectively preventing the development of sedentary behaviour in 9 to 11-year-old children.
